# Bone Strength/Bone Mass Discrepancy in Glucocorticoid‐Treated Adult Mice

**DOI:** 10.1002/jbm4.10443

**Published:** 2020-12-21

**Authors:** Alanna M. Dubrovsky, Jeffrey S. Nyman, Sasidhar Uppuganti, Kenneth J. Chmiel, Donald B. Kimmel, Nancy E Lane

**Affiliations:** ^1^ Center for Musculoskeletal Health University of California at Davis Medical Center Sacramento CA USA; ^2^ Department of Orthopaedic Surgery Vanderbilt University Medical Center Nashville TN USA; ^3^ Department of Physiological Sciences University of Florida Gainesville FL USA

**Keywords:** 6‐METHYLPREDNISOLONE, BONE HYDRATION, CORTICAL BONE, ^1^H‐NMR RELAXOMETRY, TRABECULAR BONE

## Abstract

Glucocorticoids increase bone fragility in patients in a manner that is underestimated by bone mass measurement. This study aimed to determine if the adult mouse could model this bone strength/bone mass discrepancy. Forty‐two 13‐week‐old BALB/cJ mice were randomized into vehicle and glucocorticoid groups, implanted with vehicle or 6‐methylprednisolone pellets, and necropsied after 60 and 120 days. Bone strength and bone mass/microarchitecture were assessed at the right central femur (CF; cortical‐bone–rich) and sixth lumbar vertebral body (LVB6; trabecular‐bone–rich). Bound water (BW) of the whole right femur was analyzed by proton‐nuclear magnetic resonance (^1^H‐NMR) relaxometry. Data were analyzed by two‐factor ANOVA with time (day 60 and day 120) and treatment (vehicle and glucocorticoid) as main effects for all data. Significant interactions were further analyzed with a Tukey's post hoc test. Most bone strength measures in the CF were lower in the glucocorticoid group, regardless of the duration of treatment, with no time × treatment interaction. However, bone mass measures in the CF showed a significant time × treatment interaction (*p* = 0.0001). Bone strength measures in LVB6 showed a time × treatment interaction (*p* < 0.02) such that LVB6 strength was lower after 120 days of glucocorticoids compared with 120 days of vehicle treatment. Whole‐femur–BW was lower with both glucocorticoid treatment (*p* = 0.0001) and time (*p* < 0.02), with a significant time × treatment interaction (*p* = 0.005). Glucocorticoid treatment of male BALB/cJ mice resulted in the lowering of bone strength in both cortical and trabecular bone that either appeared earlier or was greater than the treatment‐related changes in bone mass/microarchitecture. The adult mouse may be a good model for investigating the bone strength/mass discrepancy observed in glucocorticoid‐treated patients. © 2020 The Authors. *JBMR Plus* published by Wiley Periodicals LLC. on behalf of American Society for Bone and Mineral Research.

## Introduction

Glucocorticoids induce osteoporosis and osteonecrosis in humans.^(^ ^1^, ^2^, ^3^, ^4^, ^5^, ^6^, ^7^ ^)^ In‐depth study of glucocorticoid‐induced osteoporosis has revealed that the reduction in bone mass incompletely explains the increased fracture risk.^(^ ^8^, ^9^, ^10^, ^11^, ^12^, ^13^ ^)^ Clinical studies of the glucocorticoid‐induced bone strength/mass discrepancy have been challenging because data that directly inform about bone mass, formation, and microarchitecture require multiple in vivo measurements of bone mass and microarchitecture, and bone biopsies that together present logistical problems.

Current data indicate that the adult mouse model of glucocorticoid‐induced osteoporosis is sufficiently reliable to evaluate its potential relevance as a model of the glucocorticoid‐induced bone strength/mass discrepancy in patients treated with glucocorticoids. Adult mouse experiments that address glucocorticoid‐induced osteoporosis administer glucocorticoids to mice aged ≥11 weeks, last 28 to 90 days, and examine data from one time point.^(^ ^14^, ^15^, ^16^, ^17^, ^18^, ^19^, ^20^, ^21^, ^22^, ^23^, ^24^, ^25^, ^26^, ^27^, ^28^, ^29^, ^30^, ^31^, ^32^, ^33^, ^34^ ^)^ The most frequent mouse strain is a C57BL6‐like background,^(^ ^16^, ^17^, ^18^, ^19^, ^20^, ^22^, ^24^, ^25^, ^26^, ^27^, ^28^, ^30^, ^31^ ^)^ but Swiss‐Webster^(^ ^21^, ^32^, ^33^, ^34^ ^)^ and FVB/N^(^ ^23^ ^)^ have also been used. There is indication that the skeleton of all strains responds negatively to glucocorticoids with varying rates and intersite variation.^(^ ^14^ ^)^ Though a few publications report both bone strength and bone mass/microarchitecture data,^(^ ^16^, ^17^, ^18^, ^19^, ^20^, ^21^, ^22^ ^)^ most report only bone mass/microarchitecture data.^(^ ^23^, ^24^, ^25^, ^26^, ^27^, ^28^, ^29^, ^30^, ^31^, ^32^, ^33^, ^34^ ^)^ The studies tend to agree in finding that bone strength and bone mass/microarchitecture are generally lower with glucocorticoid treatment. Though the existing studies are promising, too few studies measure both bone strength/bone mass at the same site to allow evaluation of the bone strength/bone mass discrepancy in patients treated with glucocorticoids. Importantly, the universal reporting of only one time point per study leaves open the possibility that bone strength and bone mass/microarchitecture evolve at different rates during glucocorticoid treatment.

Fracture risk rises early and significantly with glucocorticoid treatment in humans.^(^ ^5^, ^8^, ^9^, ^12^ ^)^ Few, if any, preclinical or clinical studies have determined the time course for loss of bone strength and bone mass with glucocorticoid treatment. Bone strength is estimated in the clinic by DXA as BMD, an endpoint that itself imprecisely predicts fracture risk reduction by antiresorptive treatment of postmenopausal osteoporosis patients.^(^ ^35^, ^36^ ^)^ Factors considered potential BMD‐independent markers of bone strength and fracture risk include bone microarchitecture, bone mineralization, bone turnover markers, advanced glycation end products,^(^ ^37^ ^)^ and bone hydration.^(^ ^38^ ^)^ The role of bone hydration in glucocorticoid‐related osteoporosis is understudied.^(^ ^39^ ^)^

In an effort to further investigate the relationship between glucocorticoid treatment and bone strength, mass, microarchitecture, and hydration, we treated adult male Balb/cJ mice with methylprednisolone for 60 or 120 days. We hypothesized that the negative effect of glucocorticoid on bone strength, mass, and other variables is established by 60 days treatment as previously shown^(^ ^14^, ^15^, ^16^, ^17^, ^18^, ^19^, ^20^, ^21^, ^22^, ^23^, ^24^, ^25^, ^26^, ^27^, ^28^, ^29^, ^30^, ^31^, ^32^, ^33^, ^34^ ^)^ and evolves during the next 60 days of treatment. We further hypothesized that bone strength is associated with bone hydration.

## Materials and Methods

### Animals

Nine‐week‐old male BALB/cJ mice were purchased (Jackson Laboratory, Sacramento, CA, USA) and acclimated for 28 days at the UC Davis animal facility. BALB/cJ mice were chosen because their skeleton responds negatively to glucocorticoids.^(^ ^40^, ^41^, ^42^ ^)^ Mice were kept singly in plastic cages with a 12‐hour:12‐hour dark:light cycle and a temperature range of 20 to 22°C. They were fed commercial rodent chow (22/5 Rodent Diet; Teklad, Madison, WI, USA) and tap water *ad libitum*. Body weight was measured weekly. Mice were randomized by body weight into groups receiving vehicle treatment (vehicle, *n* = 22) or glucocorticoid pellets (6‐methylprednisolone, 2.5 mg 21d pellet; catalog #G‐241, Innovative Research of America, Sarasota, FL, USA; *n* = 20). All pellets were replaced on days 21, 41, 61, 81, and 101 of the study. Mice were necropsied after 60 (vehicle, *n* = 11; glucocorticoid, *n* = 9) or 120 days (vehicle, *n* = 11; glucocorticoid, *n* = 11). The study was carried out following recommendations in the Guide for the Care and Use of Laboratory Animals of the National Institutes of Health with the approval of the UC Davis Institutional Animal Care and Utilization Committee.

### Necropsy

Mice were euthanized by CO_2_ asphyxiation, followed by cardiac puncture. Cervical dislocation was used as a secondary method of euthanasia. The right femur was dissected free at the acetabulum and separated from the tibia. Lumbar vertebrae (LV) 3 to 6 were dissected free from the other vertebrae and sacrum. The right femur and the LV segment were cleansed gently of attached muscle, wrapped in saline‐soaked gauze, and frozen at −20°C.

### Biomechanical testing

#### Femoral diaphysis (three‐point bending)

After proton nuclear magnetic resonance (^1^H‐NMR) relaxometry and μCT (see below), three‐point bending tests of hydrated right femurs were performed using a servohydaulic material testing system (Instron DynaMight 8841; Instron, Norwood, MA, USA) fitted with a linear variable displacement transducer (attached to the actuator) and a 100‐N load cell (model #060‐C863‐02; Honeywell, Columbus, OH, USA). The whole femur was thawed at room temperature in PBS for 2 hours prior to testing. Femoral length and femoral diameter at the midpoint (anterior–posterior width) were measured with digital calipers. The whole femur was placed anterior surface down and medial side forward with a lower span of 8 mm, and then loaded to failure at its midpoint, at 3 mm/min. Customary biomechanical properties of bone were determined from the load‐deformation curve.^(^ ^43^, ^44^ ^)^ For example, ultimate bending moment is ultimate load times the span divided by 4, and bending strength (ultimate stress) is ultimate bending moment divided by section modulus.^(^ ^43^, ^45^ ^)^

#### Lumbar vertebral body 6 (compression)

The lumbar vertebral body 6 (LVB6) was tested in axial compression. LVB6 was dissected free from LV3 to LV5. Posterior elements and transverse processes were trimmed from each LVB6 behind the pedicle; endplates were cut parallel using a scalpel blade under water irrigation. Samples were placed between rigid stainless‐steel–loading platens with a moment relief, and then compressed to failure at 0.05 mm/s using the above‐materials–testing system. Customary measurements of bone strength were calculated from the load‐deformation curve, including the area under the curve until ultimate load (work to failure). The estimates of yield and ultimate strength were yield and ultimate load divided by the cross‐sectional bone area, which is the segmented bone volume divided by the axial length between the endplates from μCT.^(^ ^43^, ^44^, ^45^ ^)^

#### Micro‐computed tomography

All scanning was done prior to mechanical testing. The LVB6 was imaged with a high‐resolution scanner (Scanco μCT50; Scanco Medical AG, Brüttisellen, Switzerland) with X‐ray energy settings of 55 kVp and 200 μA, a 0.5‐mm Al filter, an integration time of 1200 ms, and acquisition of 1024 samples per 1000 projections over a full rotation of the sample tube. Upon reconstruction, this provided an isotropic voxel size of 12 μm. The right central femur (CF), a cortical‐bone–rich region and right distal femur were imaged with the following settings: 70 kVp and 114 μA, a 0.1‐mm Al filter, an integration time of 300 ms, and acquisition of 1024 samples per 1000 projections over a full rotation. Upon reconstruction, these settings provided an isotropic voxel size of 6 μm. Distal femur scanning was initiated 1‐mm proximal to the distal end of the femur and extended proximally for 620 slices. CF scanning was initiated 0.6‐mm proximal to the midpoint of the femur, where the bone is maximally loaded during the three‐point bending test and extended distally for 120 slices. LVB6 scanning was initiated at its cranial end and continued to its caudal end, ensuring inclusion of the entire region between the endplates (240 slices). It was ensured during sample setup that the long axis of the bones was aligned with the scanning axis of the μCT tube holder. 3D reconstruction of each scan was done with the manufacturer's software, applying a beam‐hardening correction for 1200 mg of hydroxyapatite (HA) per cm^3^. Routine scans of a HA phantom allowed the X‐ray attenuation values to be converted to volumetric mineral density. For the distal femur, a trabecular volume of interest (VOI) with a boundary 0.3‐mm proximal to the distal‐most point of the growth cartilage–metaphyseal junction, was selected and extended 450 slices. For LVB6, only the trabecular bone region between the endplates of the vertebral body was analyzed. After contouring each axial slice of the VOI to isolate the trabecular compartments, the manufacturer's evaluation algorithm was used to calculate bone volume fraction (BV/TV), trabecular thickness (Tb.Th), trabecular number (Tb.N), trabecular spacing (Tb.Sp), and structure model index (SMI), and cross‐sectional bone area (Tt.Ar), as described previously.^(^ ^46^ ^)^ The bone segmentation parameters in this study were Gaussian sigma = 0.2, support = 1.0, and a global threshold = 538.2 mg HA/cm^3^ for Distal femur (DFM) and Gaussian sigma = 0.2, support = 1.0, and a global threshold = 474.7 mg HA/cm^3^ LVB6.^(^ ^46^, ^47^, ^48^ ^)^

For the right CF, the 100 slices surrounding the midpoint of the femur were considered as the CF VOI. After fitting contour lines to the periosteal and endosteal surfaces of all the reconstructed images, we again used the manufacturer's standard evaluation script to determine cortical area (Ct.Ar), total area (Tt.Ar), cortical area/total area (Ct.Ar/Tt.Ar), and cortical thickness (Ct.Th). The segmentation or thresholding parameters were Gaussian sigma = 0.2, support = 1.0, and a global threshold = 828.1 mg HA/cm^3^. From Euler‐Bernoulli beam theory, the ultimate moment is directly proportional to the cross‐sectional geometry factor known as the section modulus (SM). SM was defined as I_min_/c_min_, where I_min_ is the minimum principal moment of inertia that corresponds to bending within the anterior–posterior plane and c_min_ corresponds to the distance between the neutral axis (zero stress) and outermost bone surface in the direction of loading.^(^ ^43^, ^44^ ^)^

#### ^1^H‐NMR relaxometry

**Fig 1:**
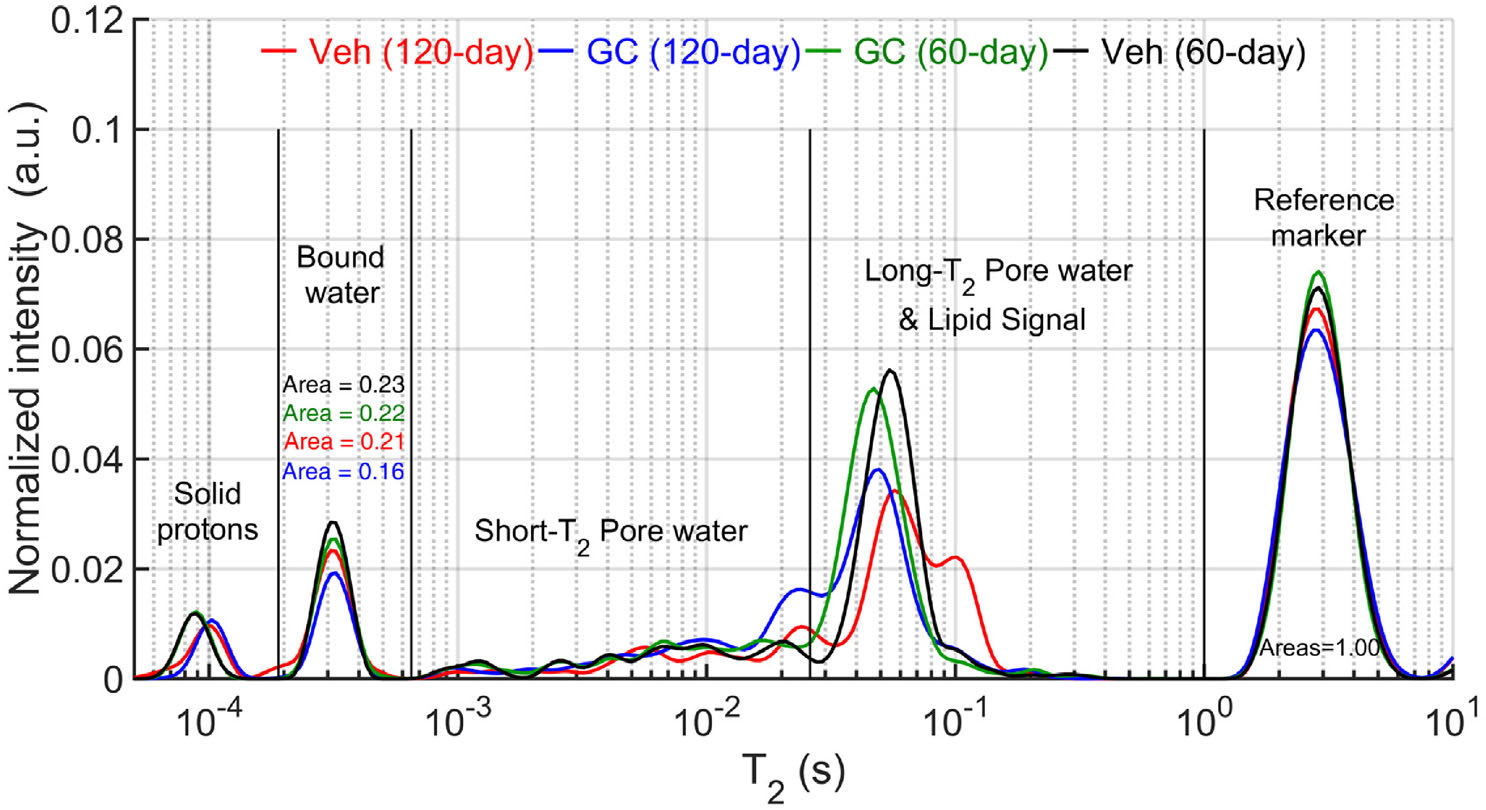
Averaged T_2_ spectra for each group. Using proton‐nuclear magnetic resonance relaxometry, the relaxation time of bound water is faster than the relaxation time of pore water, allowing separation and quantification of the two pools. Because signals from lipids overlap signals from water in large pores, the integration of the area was shortened (indicated by vertical lines). The signal intensities were normalized by signals from the reference marker such that the area under the marker curve was equal to 1.00. The bound‐water peak was lower for the glucocorticoid‐treated groups than for the vehicle‐treated groups.

Approximately 25% of bone is composed of water existing freely in vascular channels (pore or mobile water) or bound to the extracellular matrix.^(^ ^49^ ^)^ Both matrix‐BW and free water are associated with flexural bone strength and work‐to‐fracture.^(^ ^50^ ^)^ Bone hydration can be measured using ^1^H‐NMR relaxometry in a nondestructive manner because the spin state of water hydrogen bonding with the matrix relaxes faster than the spin state of pore water (PW). By including a reference marker of water with a slow relaxation rate next to the bone, volume fractions of BW and PW were measured on intact right femurs following our previously described ^1^H‐NMR relaxometry technique.^(^ ^43^, ^51^ ^)^ Briefly, the femur and reference marker were placed inside a custom‐built radiofrequency coil,^(^ ^52^ ^)^ which was subsequently inserted into a 4.7‐T horizontal bore magnet (Varian Medical Systems, Santa Clara, CA, USA). Carr‐Purcell‐Meiboom‐Gill measurements were fit with exponential functions to generate a T_2_ spectrum (Fig. 1). The normalized signals of the matrix‐BW pool and the PW pool were converted to volume fractions as a percentage of water volume within specimen bone volume, which was measured by Archimedes' principle.^(^ ^38^ ^)^

### Statistical Analysis

Group means and standard deviations were calculated for all variables, and Pearson's correlations were performed between bone strength variables and BW (GraphPad Prism 7.00; GraphPad Software, La Jolla, CA, USA). Two‐factor ANOVA was performed to determine the effects of glucocorticoid treatment and treatment time. Differences were considered significant at *p* < 0.05. If the interaction between treatment and time was significant, Tukey's post hoc test was used to determine whether there were significant differences.

## Results

### Effect of glucocorticoid treatment

#### Anthropometric and bone hydration endpoints

Change in body weight was negatively affected by glucocorticoid treatment (*p* < 0.0027; Table 1). Femoral BW was lower and femoral PW was greater in glucocorticoid‐treated mice than vehicle‐treated mice (Table 1). These glucocorticoid effects depended on treatment time (ie, significant interaction), and in post hoc multiple‐comparison tests, BW decreased and PW increased with glucocorticoid treatment but not with vehicle treatment.

**Table 1:** Femur Hydration, Central Femur Biomechanical Properties, and Morphometry

Variable	Units	Vehicle day 60	Glucocorticoid day 60	Vehicle day 120	Glucocorticoid day 120	Effect of time	Effect of treatment	Interaction
		Mean ± SD	Mean ± SD	Mean ± SD	Mean ± SD	*p*	*p*	*p*
Change in body weight	g	4.17 ± 2.69	1.62 ± 2.31	3.86 ± 0.84	2.11 ± 2.13	0.8971	0.0027	0.5483
Hydration		N = 11	N = 11	N = 9	N = 11			
Femur bound water	%	10.05 ± 0.68	9.21 ± 0.62^a^	10.13 ± 0.84	8.05 ± 0.56^a^ ^,^ ^b^	0.0139	0.0001	0.0050
Femur pore water	%	11.77 ± 1.06	13.77 ± 1.66^a^	10.84 ± 1.15	17.67 ± 2.03^a^ ^,^ ^b^	0.0037	0.0001	0.0001
Mechanical properties		N = 11	N = 10	N = 9	N = 10			
Bending strength (ultimate stress)	MPa	200.4 ± 29.5	158.6 ± 32.0	185.4 ± 41.1	141.7 ± 22.2	0.1211	0.0002	0.9255
Yield strength (yield stress)	MPa	186.1 ± 21.5	151.2 ± 28.0	175.6 ± 34.0	124.1 ± 28.9	0.0416	0.0001	0.3609
Ultimate moment	N mm	39.08 ± 6.26	30.38 ± 7.58	38.83 ± 9.41	24.92 ± 4.36	0.2103	0.0001	0.2520
Yield moment	N mm	36.28 ± 4.52	28.92 ± 6.66	36.75 ± 7.87	21.68 ± 5.05	0.0869	0.0001	0.0527
Ultimate load	N	19.29 ± 2.86	15.18 ± 3.80	19.42 ± 4.71	12.47 ± 2.18	0.2479	0.0001	0.2039
Yield load	N	16.08 ± 1.98	13.00 ± 2.70^a^	17.19 ± 3.45	10.11 ± 1.58^a,^	0.2654	0.0001	0.0158
Work to failure	MPa	4.26 ± 1.90	2.32 ± 1.51	3.38 ± 2.28	1.78 ± 0.76	0.1939	0.0023	0.7599
Modulus	MPa	12.27 ± 1.36	10.40 ± 1.61	12.42 ± 1.89	8.57 ± 1.77	0.1164	0.0001	0.0667
Stiffness	N/mm	125.3 ± 16.6	106.6 ± 11.9	141.6 ± 22.7	84.7 ± 18.9^a^ ^,^ ^b^	0.6258	0.0001	0.0018
Morphometry		N = 11	N = 11	N = 9	N = 11			
Cortical area	mm^2^	0.958 ± 0.045	0.869 ± 0.055^a^	1.025 ± 0.044^b^	0.774 ± 0.042^a^ ^,^ ^b^	0.3577	0.0001	0.0001
Cortical area/total area	‐	0.571 ± 0.018	0.503 ± 0.011^a^	0.586 ± 0.014	0.447 ± 0.024^a^ ^,^ ^b^	0.0008	0.0001	0.0001
Cortical thickness	mm	0.197 ± 0.005	0.182 ± 0.005^a^	0.210 ± 0.005^b^	0.172 ± 0.010^a^ ^,^ ^b^	0.4900	0.0001	0.0001
Femoral length	mm	14.63 ± 0.26	14.08 ± 0.22	14.85 ± 0.50	14.11 ± 0.38	0.2708	0.0001	0.3932
Diameter	mm	1.11 ± 0.02	1.13 ± 0.03	1.11 ± 0.04	1.15 ± 0.06	0.3920	0.0207	0.7452
Total area	mm^2^	1.676 ± 0.055	1.730 ± 0.096	1.748 ± 0.050	1.734 ± 0.068	0.0867	0.3597	0.1254

^a^ Significant difference between vehicle and glucocorticoid treatment within each time group (Tukey's adjusted *p* < 0.05).

^b^ Significant difference between day 60 and day 120 within each treatment group (Tukey's adjusted *p* < 0.05).

#### Central femur mechanical properties

Bending strength measurements, ultimate and yield stress, were 21% and 19% lower, respectively, in mice treated with glucocorticoid for 60 days than in vehicle‐treated mice (Table 1). Ultimate moment and yield moment were 22% and 20% lower, respectively, and ultimate load and yield load were 23% and 19% lower, respectively, in glucocorticoid‐treated mice (60 days) than vehicle‐treated mice (Table 1). These glucocorticoid effects on the biomechanical properties of the central diaphysis persisted after 120 days of treatment but did not appear to progressively worsen with prolonged exposure to glucocorticoid (nonsignificant interaction). Regardless of treatment time, work to failure was 45% lower. Modulus and stiffness were also lower in glucocorticoid‐treated mice than vehicle‐treated mice (Table 1). The glucocorticoid effect on stiffness, however, depended on the treatment time because stiffness was significantly lower after glucocorticoid treatment for 120 days compared with glucocorticoid treatment for 60 days.

#### Central femur morphometry

Ct.Ar, Ct.Ar/Tt.Ar, and Ct.Th were all lower in glucocorticoid‐treated mice compared with vehicle‐treated mice (Table 1), and these glucocorticoid treatment effects depended on the treatment time (Table 1) as Ct.Ar and Ct.Th increased between day 60 and day 120 with vehicle treatment but decreased between day 60 and day 120 with glucocorticoid treatment (Table 1).

#### LV body biomechanical properties and morphometry

Glucocorticoid treatment affected the lumbar body ultimate load and work to ultimate force in compression, but the interaction between treatment and duration of treatment was significant such that these biomechanical properties of the vertebral body were lower after 120 days of glucocorticoid treatment but not after 60 days of glucocorticoid treatment. Interestingly, glucocorticoid treatment did not affect trabecular BV/TV (Table 2). There was only a significant effect on SMI with trabeculae becoming more rod‐like with glucocorticoid treatment.

**Table 2:** Lumbar Vertebral Body Biomechanical Properties and Morphometry

Variable	Units	Vehicle day 60	Glucocorticoid day 60	Vehicle day 120	Glucocorticoid day 120	Effect of time	Effect of treatment	Interaction
		Mean ± SD	Mean ± SD	Mean ± SD	Mean ± SD	*p*	*p*	*p*
Mechanical properties		N = 11	N = 10	N = 7	N = 11			
Ultimate load	N	20.92 ± 3.48	22.72 ± 3.61	24.34 ± 4.53	15.56 ± 3.64^a^ ^,^ ^b^	0.0595	0.0157	0.0004
Yield load	N	16.85 ± 4.14	19.38 ± 4.48	20.43 ± 5.48	12.07 ± 2.73^a^ ^,^ ^b^	0.0455	0.0913	0.0007
Work to ultimate force	N mm	2.94 ± 1.42	2.97 ± 1.68	4.33 ± 2.09	1.90 ± 1.16^a,^	0.7402	0.0262	0.0223
Yield strength	MPa	29.75 ± 9.25	33.76 ± 7.78	33.18 ± 10.16	22.18 ± 4.05^b^	0.0309	0.3727	0.0148
Ultimate strength	MPa	36.56 ± 6.90	39.93 ± 8.51	39.50 ± 8.82	28.56 ± 4.95^b^	0.0338	0.2139	0.0099
Morphometry		N = 11	N = 11	N = 8	N = 11			
Bone volume (BV/TV)	%	22.27 ± 2.02	23.93 ± 3.49	20.68 ± 1.87	18.79 ± 3.53	0.0008	0.9009	0.0610
Structure model index	—	0.858 ± 0.233	0.939 ± 0.219	0.968 ± 0.162	1.393 ± 0.408	0.0028	0.0066	0.0576
Trabecular number	mm^−1^	5.09 ± 0.22	5.02 ± 0.26	4.71 ± 0.38	4.82 ± 0.30	0.0036	0.8450	0.3161
Trabecular thickness	mm	0.046 ± 0.003	0.050 ± 0.006	0.046 ± 0.001	0.044 ± 0.003	0.0183	0.4886	0.0538
Trabecular spacing	mm	0.200 ± 0.010	0.203 ± 0.012	0.219 ± 0.018	0.209 ± 0.014	0.0058	0.5249	0.1446

^a^ Significant difference between vehicle and glucocorticoid treatment within each time group (Tukey's adjusted *p* < 0.05).

^b^ Significant difference between day 60 and day 120 within each treatment group (Tukey's adjusted *p* < 0.05).

### Effect of treatment time

#### Anthropometric and bone hydration endpoints

Change in body weight was not affected by duration of treatment (*p* = 0.8971; Table 1). Femoral BW on the other hand was negatively affected by both glucocorticoid treatment (*p* = 0.0001) and duration of treatment (*p* = 0.0139), while femoral PW was positively affected by both glucocorticoid treatment (*p* = 0.0001) and duration of treatment (*p* = 0.0037).

#### Central femur mechanical properties

Bending strength (ie, ultimate stress), ultimate moment, yield moment, modulus, and work to failure were not affected by treatment duration, and the interaction between treatment and duration of treatment was not significant (Table 1). Yield strength was modestly affected by treatment duration (*p* = 0.042), but showed no interaction between treatment and duration of treatment. Interestingly, whole‐femur–BW was significantly correlated with ultimate bending moment of the CF at day 60 (Pearson *r* = 0.7387, *p* = 0.0001) and day 120 (Pearson *r* = 0.7002, *p* = 0.0008).

#### Central femur morphometry

Ct.Ar and Ct.Th were not affected by duration of treatment, but there was an interaction between treatment and duration (*p* = 0.0001). Ct.Ar/Tt.Ar was lower with increased duration of treatment (*p* = 0.0008). Femur length and diameter were negatively affected by glucocorticoids (*p* = 0.0001 and *p* = 0.0207, respectively) but not by treatment duration. There was no interaction between treatment and duration.

#### LV body biomechanical properties and morphometry

Ultimate strength and yield strength were affected by duration of treatment (Table 2). Both showed significant interactions between treatment and duration (*p* = 0.0148 and *p* = 0.0099, respectively). In other words, these measures of compressive strength, normalized to Tt.Ar of the vertebral body, were significantly lower after glucocorticoid treatment for 120 days compared to glucocorticoid treatment for 60 days, whereas there was no significant change with time in the vehicle group. Bone volume (BV/TV), Tb.N, and Tb.Th were lower with longer treatment time, when accounting for the treatment effect (Table 2). Both Tb.Sp and SMI increased with duration of treatment; only SMI was higher with glucocorticoid treatment (*p* = 0.0066) with no significant interaction of treatment and time.

#### Distal femur morphometry

Only Tb.Th was negatively affected by glucocorticoid treatment (*p* = 0.0001) and duration of treatment (*p* = 0.0001). Because the interaction between treatment and duration was also significant (*p* = 0.0270), the negative effect of glucocorticoid on Tb.Th depended on the duration. Between day 60 and day 120, Tb.Th decreased only in the glucocorticoid‐treated mice. No other trabecular parameters were affected by either glucocorticoid or duration of treatment (Table 3).

**Table 3:** Distal Femur Morphometry

Variable	Units	Vehicle day 60	Glucocorticoid day 60	Vehicle day 120	Glucocorticoid day 120	Effect of time	Effect of treatment	Interaction
		Mean ± SD	Mean ± SD	Mean ± SD	Mean ± SD	*p*	*p*	*p*
		N = 10	N = 9	N = 9	N = 11			
Bone volume (BV/TV)	%	11.76 ± 1.47	12.33 ± 1.29	12.18 ± 1.39	10.70 ± 1.45	0.1877	0.3243	0.0297
Structure model index	—	1.94 ± 0.16	1.87 ± 0.13	1.80 ± 0.16	2.03 ± 0.13^a^	0.7826	0.1023	0.0029
Trabecular number	mm^−1^	4.95 ± 0.27	5.24 ± 0.42	5.00 ± 0.45	5.12 ± 0.43	0.8044	0.1138	0.4869
Trabecular thickness	mm	0.037 ± 0.002	0.034 ± 0.001^a^	0.035 ± 0.002^ns^	0.031 ± 0.001^a^ ^,^ ^b^	0.0001	0.0001	0.0270
Trabecular spacing	mm	0.201 ± 0.011	0.192 ± 0.016	0.199 ± 0.020	0.195 ± 0.019	0.9153	0.2058	0.6176

ns, not significant.

^a^ Significant difference between vehicle and glucocorticoid treatment within each time group (Tukey's adjusted *p* < 0.05).

^b^ Significant difference between day 60 and day 120 within each treatment group (Tukey's adjusted *p* < 0.05).

## Discussion

The glucocorticoid‐treated group had lower cortical bone strength both with respect to structural‐dependent properties and material property estimates, regardless of the duration of treatment, paralleling data from previous investigations of these two endpoints in glucocorticoid‐treated mice.^(^ ^16^, ^17^, ^18^ ^)^ Others reported a glucocorticoid‐related difference in cortical bone mass only without reporting cortical bone strength data.^(^ ^20^, ^22^, ^23^, ^24^, ^25^ ^)^ On the other hand, there was no glucocorticoid effect on either ultimate compressive stress or trabecular bone volume in the LVB6 or trabecular bone volume in the distal femoral metaphysis at day 60 of treatment, as reported previously.^(^ ^16^, ^26^ ^)^ However, others have reported significantly lower trabecular bone mass with glucocorticoid treatment in this timeframe.^(^ ^17^, ^18^, ^19^, ^21^, ^22^, ^23^, ^24^, ^25^, ^26^, ^28^, ^29^, ^31^, ^32^, ^33^, ^34^ ^)^ We conclude from these day 60 data that cortical bone of the Balb/CJ mouse, like that of other strains, responds negatively to glucocorticoids, whereas trabecular bone is less affected.

Most indices of cortical bone strength displayed a negative effect of glucocorticoid treatment by day 60 that did not worsen by day 120. A few studies have noted that glucocorticoid‐related differences in cortical bone strength in mice emerge as early as 28 days.^(^ ^16^, ^17^, ^18^ ^)^ Because our earliest time point was day 60, our data leave open the possibility that the glucocorticoid‐related differences in cortical bone strength plateau as early as 28 days of treatment. A direct comparison of bone strength after 28 and 60 days of glucocorticoid treatment in adult mice is needed to establish whether day 28 represents a plateau phase of glucocorticoid effects on cortical bone strength.

The major indices of cortical bone mass and architecture, Ct.Ar, Ct.Ar/Tt.Ar, and Ct.Th, showed an effect of glucocorticoid treatment and an interaction of glucocorticoid treatment with duration of treatment. This indicates both that the glucocorticoid‐related bone mass/micoarchitecture difference was significantly greater at day 120 than at day 60 and that cortical bone loss continued after the decline in cortical bone strength seemingly plateaued. The one exception was yield strength (time, *p* = 0.0416). Because our experiment ended at day 120, we cannot be certain that bone mass changes had plateaued in Balb/cJ male mice. An informative experiment at this point would be to begin glucocorticoid treatment in 15‐week‐old C57BL6 male or female mice and necropsy at time points that include at least days 28 to 180 to both match existing studies and add longer times that thoroughly investigate when new steady states of bone strength and mass are achieved in glucocorticoid‐treated mice. Given the known effects of glucocorticoids on body weight, some form of pair‐feeding to match body weight of glucocorticoid‐treated and vehicle‐treated mice should be considered. Cortical and trabecular bone sites should be assessed for strength, mass, microarchitecture, turnover, and other measures of bone quality. Such a serial sacrifice experiment is needed for glucocorticoid treatment because the relationship of bone strength to bone mass with glucocorticoid treatment differs from age‐related bone loss.^(^ ^8^ ^)^ A similar time‐course experiment was performed in the ovariectomized rat^(^ ^53^, ^54^, ^55^ ^)^ to reveal when estrogen‐deficiency bone loss and turnover plateaued. The early knowledge of this time course resulted in a robust model that has been used hundreds of times to test the effect of medications to either prevent or treat established osteoporosis.^(^ ^56^, ^57^ ^)^

The changes in the bone strength and mass at the vertebral body in response to glucocorticoid exposure were not present until day 120, but the treatment‐related decline was only significant for VB strength. Specifically, at day 120, ultimate load was significantly lower in the glucocorticoid‐treated mice than in the vehicle‐treated mice. This was not the case for BV/TV. Interestingly, the change in bone strength appeared later in trabecular bone than in cortical bone. Three prior mouse experiments reported glucocorticoid‐related differences in trabecular bone strength after 28 days,^(^ ^18^, ^20^, ^22^ ^)^ whereas others found no difference at even longer times.^(^ ^21^ ^)^ Our data leave open the chance that the glucocorticoid‐related difference in trabecular bone strength continues to evolve after day 120. A study duration greater than 120 days is needed to establish when reduced trabecular bone strength caused by glucocorticoid‐treatment plateaus, and if and when trabecular bone mass and microarchitecture are clearly affected by glucocorticoid treatment in the Balb/cJ mouse, as has been previously reported.^(^ ^17^, ^18^, ^19^, ^21^, ^22^, ^23^, ^24^, ^25^, ^26^, ^28^, ^29^, ^31^, ^32^, ^33^, ^34^ ^)^ Trabecular bone strength declined without significant changes in trabecular bone mass. These findings point to the likelihood that non‐bone‐mass properties that affect bone strength in both cortical and trabecular bone deteriorate before bone mass, suggesting that identification of those properties could encourage attempts to affect them, perhaps preventing loss of bone strength caused by glucocorticoid treatment.

Small animal models of glucocorticoid‐induced osteopenia in adult humans are less consistent than those for estrogen‐deficiency osteopenia.^(^ ^54^, ^55^, ^56^, ^57^, ^58^, ^59^, ^60^ ^)^ Considering the variety of glucocorticoid‐dosing options and mouse strains, and choice of experimental durations that may be based on convenience rather than glucocorticoid pharmacology and physiology, this is not surprising. Yet enough consensus can be found among existing studies of glucocorticoid‐treated adult mice^(^ ^14^, ^15^, ^16^, ^17^, ^18^, ^19^, ^20^, ^21^, ^22^, ^23^, ^24^, ^25^, ^26^, ^27^, ^28^, ^29^, ^30^, ^31^, ^32^, ^33^, ^34^ ^)^ to consider studying the puzzling bone strength/bone mass discrepancy in patients treated with glucocorticoids.^(^ ^8^, ^11^ ^)^ Measuring cortical and trabecular bone strength and bone mass endpoints at multiple necropsy periods in the same anatomical site appears to have revealed an opportunity. The treatment‐related asynchronous decline in bone strength and bone mass was different between cortical and trabecular bone; the decline in VB strength depended on a long duration of glucocorticoid treatment and the decline in bending strength of the CF diaphysis occurred early. The decline in ultimate stress, an estimate of material strength, with treatment possibly suggests a bone mass‐independent role of additional bone quality factors in determining bone strength could be studied preclinically.^(^ ^37^ ^)^ In addition, the decline in bone strength that precedes the decline in bone mass has fundamental parallels to clinical observations in many glucocorticoid patients during the first year of treatment.^(^ ^8^ ^)^

A non‐bone‐mass property related to bone strength that we studied was bone hydration. The glucocorticoid group showed lower whole‐femur–BW at day 60 that continued to worsen by day 120. Our findings on the relationship of bone hydration to bone strength concur with earlier data.^(^ ^39^ ^)^ The continued decrease in BW between days 60 and 120, a period when cortical bone strength did not change, is a bit perplexing. However, we noted that trabecular‐rich vertebral body strength declined between days 60 and 120 and that our hydration measure, whole‐femur–BW, reflects a contribution from the trabecular bone found in the distal and proximal metaphyses of the femur. Also, whole‐femur–BW was significantly correlated with the ultimate bending moment of the CF at day 60 and day 120. The glucocorticoid‐related decrease in BW may be because of a decline in blood supply to bone. Treating mice with antivascular endothelial growth factor A monoclonal antibody for 42 days decreased both BW of the whole femur and distal femur blood flow.^(^ ^45^ ^)^ As another possibility, glucocorticoid treatment could modify the organic matrix of bone in a way that reduces the hydrogen bonding between water and amino acids that gives rise to BW. In a mouse model of glucocorticoid‐induced osteoporosis (*Crh* ^*‐120/+*^), anterior femoral strips from mice with endogenous hypercorticosteronaemia experienced higher fibril strain for a given applied tensile stress than did similar femoral strips from WT mice.^(^ ^62^ ^)^ In effect, these synchrotron X‐ray diffraction tests revealed that glucocorticoid excess significantly decreases the effective fibril modulus and D spacing of the collagen I fibril, suggesting exogeneous glucocorticoids could modify the molecular structure of type I collagen. Future studies using mass spectrometry or other matrix characterization techniques are needed to determine whether glucocorticoids cause a decline in proteins from bone and/or identify determinant modifications to collagen I or noncollagenous proteins that reduce matrix hydration and overall material quality of the organic matrix.

This study has a number of strengths. We assessed cortical and trabecular bone strength and mass/microarchitecture in glucocorticoid‐treated BALB/cJ male mice at two times. BALB/cJ mice are a good model for glucocorticoid‐induced osteonecrosis of the distal femoral epiphysis,^(^ ^40^, ^41^, ^42^ ^)^ indicating the ability of the Balb/cJ skeleton to respond to glucocorticoids. We evaluated both bone strength and bone mass in a region composed only of cortical bone (CF) and an area composed mainly of trabecular bone (lumbar spine). We evaluated bone hydration by a state‐of‐the‐art method, ^1^H‐NMR relaxometry. However, there were a number of shortcomings. We studied only one mouse strain and one gender, so we advise caution in generalizing our data to other mouse strains or to female mice. Other studies of glucocorticoid exposure in mice have shown strain differences in the bone response to glucocorticoids.^(^ ^63^ ^)^ We did not study a baseline group of mice matched on age and sex; this limited our ability to determine significant predictors of the changes in bone mass and strength independent of glucocorticoids. The mouse does not undergo intracortical remodeling and because glucocorticoid‐induced osteoporosis can include intracortical remodeling, our results cannot be generalized to glucocorticoid‐treated subjects. Also, patients are treated with glucocorticoids to control inflammatory, noninfectious diseases, such as systemic lupus erythematosus (SLE) or vasculitis, and thus may have significant comorbidities. Our study used healthy adult male mice with no known comorbid diseases, creating potential difficulty in translating these data to patients. Additional studies in mice with an underlying inflammatory disease, such as SLE or inflammatory arthritis, may provide a more relevant model from which to translate the findings of glucocorticoids on bone to a clinical population.^(^ ^4^, ^64^ ^)^ Although the regions of interest for cortical bone strength, Ct.Ar, Ct.Th, and hydration overlap, the bone hydration measure included the entire femur, meaning that we cannot be sure that the value for whole‐femur BW value reflects bone hydration specifically in the CF region in which cortical bone strength was measured. We are currently unable to measure bone hydration in either the regionalized femur or the vertebral body at this time with ^1^H‐NMR relaxometry.

In summary, glucocorticoid treatment of male BALB/cJ mice resulted in the lowering of bone strength in both cortical and trabecular bone that either appeared later or was greater than the treatment‐related changes in bone mass/microarchitecture. With glucocorticoid treatment, bone strength in cortical bone of the femur deteriorated more rapidly than bone strength in the vertebral body. These data suggest that during glucocorticoid treatment, significant non‐bone‐mass determinants of bone strength change, implying that a greater understanding of such properties may allow for therapies to prevent those changes and ultimately reduce bone fragility caused by glucocorticoids. In addition, the adult mouse may be a good model for studying the bone strength/bone mass discrepancy seen with glucocorticoid treatment in humans.

## Disclosures

AMKD, JSN, SU, KC, DBK, and NEL hereby state that they have no conflicts of interest.

### PEER REVIEW

The peer review history for this article is available at https://publons.com/publon/10.1002/jbm4.10443.
